# The complete chloroplast genome sequence of *Bambusa albolineata* (bambusodae)

**DOI:** 10.1080/23802359.2021.1967798

**Published:** 2021-08-24

**Authors:** Zhiwen Deng, Qian Chen, Jun Wu, Ke Ren, Muhammad Waqqas Khan Tarin, Tianyou He, Lingyan Chen, Liguang Chen, Ahsan Ul Haq, Yushan Zheng

**Affiliations:** a College of Forestry, Fujian Agriculture and Forestry University, Fuzhou, PR China; b College of Landscape & Architecture, Fujian Agriculture and Forestry University, Fuzhou, Fujian350002, China; c Department of Forestry, Range Management and Wildlife, University of Agriculture Faisalabad, Pakistan

**Keywords:** *Bambusa albolineata*, plastid genome, phylogenetic analysis

## Abstract

*Bambusa albolineata* (local name: Hua Zhu) is found in Zhejiang, Jiangxi, Fujian, Taiwan, and Guangdong provinces of China, and is often cultivated on low hills, flatlands, and along streams and rivers. Due to its long internodes and flexible material, it is used as timber wood in China. In the current research, the complete chloroplast (CP) genome of *B. albolineata* was sequenced and reported for the first time. The complete CP genome sequence was 139,326 bp, including a large single-copy (LSC) region of 82,862 bp, a small single-copy (SSC) region of 12,870 bp, and a pair of invert repeats (IR) regions of 21,798 bp. Besides, the plastid genome consisted of 129 genes; having 82 protein-coding genes, 39 tRNA genes, and eight rRNA genes. The overall GC content of the genome was 44.2%. The phylogenetic analysis based on the complete chloroplast genome indicates that *B. albolineata* is strongly related to *B. flexuosa* and *B. boniopsis.*

*Bambusa albolineata* belongs to Poaceae family. The pole height is about 6–8 m with a diameter of 3.5–5.5 cm and a recurved tail tip. The internodes of this bamboo are 40–60 cm long and the poles are green with white or yellow-white longitudinal stripes. It is found in Zhejiang, Jiangxi, Fujian, Taiwan, and Guangdong provinces of China, and is often cultivated on low hills, flatlands, and along streams and rivers. *B. albolineata* has long internodes and flexible material, which is excellent for making various kinds of bamboo ware (http://www.iplant.cn/). To date, no studies have been conducted on the genome of *B. albolineata*. The chloroplasts (CP) genome has an ancestral history and a conserved structure that has been used to discover the phylogenetic and developmental relationship in plants (Wang et al. 2018). For this reason, we identified and characterized the complete CP genome sequence of *B. albolineata* by next-generation sequencing (NGS) technology in the present work. We collected the fresh leaves tissues of *B. albolineata* from Bamboo Garden, Fujian Agriculture and Forestry University, Fuzhou, Fujian province, China (119°14′6″E, 26°5′7″ N), and dried them into silica gel immediately. The specimens have been preserved in the Herbarium of College of Forestry, Fujian Agriculture and Forestry University (specimen code #HTY011).

The extraction of DNA from the fresh leaves was done following a modified CTAB method as described by Murray and Thompson (1980) and the Nextera XT DNA Library Preparation Kit was used further to establish the library with an average length (350 bp). Illumina Novaseq platform was used for the high-throughput sequencing and 150 bp was the average length of the generated reads. By using the NGS QC Tool Kit v2.3.3, Illumina raw sequence reads were edited following Ge et al. (2018) and using *de novo* assembler SPAdes3.11.0, the high-quality reads were assembled into contigs as described by Bankevich et al. (2012).

The complete CP genome sequence of *B. albolineata* has been submitted to GenBank under the accession number: MW557324 and raw reads have been deposited in the GenBank Sequence Read Archive (SRA: SRR13627099). The complete plastid genome sequence of *B. albolineata* was 139,326 bp in full-length, with a large single-copy (LSC) region of 82,862 bp, a small single-copy (SSC) region of 12,870 bp, and a pair of inverted repeats (IR) regions of 21,798 bp. The complete chloroplastid genome contained 129 genes, including 82 protein-coding genes, 39 tRNA genes, and eight rRNA genes. The complete genome GC content was 44.2%. In order to investigate the phylogenetic position of *B. albolineata* with other Bambusa members, we conducted a phylogenetic analysis based on 16 complete cp genomes of Bambusa, and one taxa (*Dendrocalamus barbatus*) as an outgroup. All of these were downloaded from NCBI GenBank. The sequences were aligned by MAFFT v7.307 following Katoh and Standley (2013), and the phylogenetic tree was generated by RAxML (Stamatakis 2014) with 1000 bootstraps replicates. The GTRGAMMA model was used in the ML analysis. In the current study, the phylogenetic analysis suggests that *B. albolineata* is similar to *B. flexuosa* and *B. boniopsis* as depicted in Figure 1.

**Figure 1.**
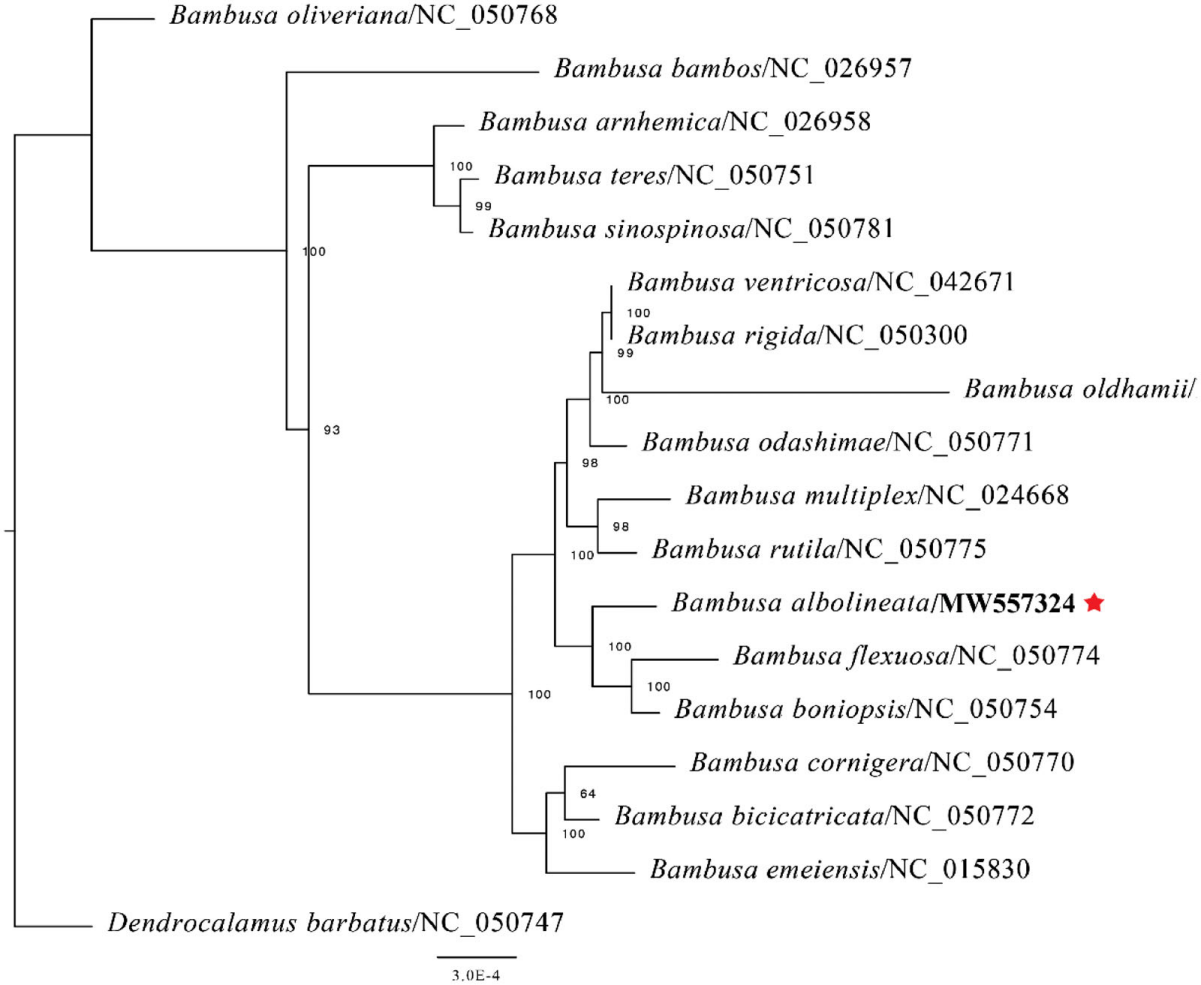
Phylogenetic analysis of 17 species of Bambusodae and one taxa (*Dendrocalamus barbatus*) as outgroup based on plastid genome sequences by RAxML, bootstrap support value near the branch.

## Data Availability

The data that support the findings of this study are openly available in GeneBank of NCBI at https://www.ncbi.nlm.nih.gov/ with the following accession number: MW557324. The associated BioProject, SRA, and Bio-Sample numbers are PRJNA699328, SRR13627099, and SAMN17774295 respectively.

## References

[CIT0001] Bankevich A, Nurk S, Antipov D, Gurevich AA, Dvorkin M, Kulikov AS, Lesin VM, Nikolenko SI, Pham S, Prjibelski AD, et al. 2012. SPAdes: a new genome assembly algorithm and its applications to single-cell sequencing. J Comput Biol. 19(5):455–477. 22506599 10.1089/cmb.2012.0021 PMC3342519

[CIT0002] Ge J, Cai L, Bi G-Q, Chen G, Sun W. 2018. Characterization of the complete chloroplast genomes of *Buddleja colvilei* and *B. sessilifolia*: implications for the Taxonomy of Buddleja L. Molecules. 23(6):1248. 10.3390/molecules23061248 PMC6100213 29882896

[CIT0003] Katoh K, Standley DM. 2013. MAFFT multiple sequence alignment software version 7: improvements in performance and usability. Mol Biol Evol. 30(4):772–780. 23329690 10.1093/molbev/mst010 PMC3603318

[CIT0004] Murray MG, Thompson WF. 1980. Rapid isolation of high molecular weight plant DNA. Nucleic Acids Res. 8(19):4321–4326. 7433111 10.1093/nar/8.19.4321 PMC324241

[CIT0005] Stamatakis A. 2014. RAxML version 8: a tool for phylogenetic analysis and post-analysis of large phylogenies. Bioinformatics. 30(9):1312–1313. 24451623 10.1093/bioinformatics/btu033 PMC3998144

[CIT0006] Wang J, Li C, Yan C, Zhao X, Shan S. 2018. A comparative analysis of the complete chloroplast genome sequences of four peanut botanical varieties. PeerJ. 6:e5349. 30083466 10.7717/peerj.5349 PMC6074784

